# Benefits of Increasing Greenness on All-Cause Mortality in the Largest Metropolitan Areas of the United States Within the Past Two Decades

**DOI:** 10.3389/fpubh.2022.841936

**Published:** 2022-05-10

**Authors:** Paige Brochu, Marcia P. Jimenez, Peter James, Patrick L. Kinney, Kevin Lane

**Affiliations:** ^1^Department of Environmental Health, Boston University School of Public Health, Boston, MA, United States; ^2^Department of Epidemiology, Boston University School of Public Health, Boston, MA, United States; ^3^Department of Population Medicine, Harvard Medical School, Harvard Pilgrim Health Care Institute, Boston, MA, United States; ^4^Department of Environmental Health, Harvard T.H. Chan School of Public Health, Boston, MA, United States

**Keywords:** greenness, all-cause mortality, built environment, United States, Normalized Difference Vegetation Index (NDVI), climate action plans

## Abstract

Across the United States, cities are creating sustainability and climate action plans (CAPs) that call to increase local vegetation. These greening initiatives have the potential to not only benefit the environment but also human health. In epidemiologic literature, greenness has a protective effect on mortality through various direct and indirect pathways. We aimed to assess how an increase in greenness could decrease mortality in the largest urban areas in the United States. We conducted a nationwide quantitative health impact assessment to estimate the predicted reduction in mortality associated with an increase in greenness across two decades (2000, 2010, and 2019). Using a recently published exposure-response function, Landsat 30 m 16-day satellite imagery from April to September, and publicly available county-level mortality data from the CDC, we calculated the age-adjusted reduction in all-cause mortality for those 65 years and older within 35 of the most populated metropolitan areas. We estimated that between 34,000 and 38,000 all-cause deaths could have been reduced in 2000, 2010, and 2019 with a local increase in green vegetation by 0.1 unit across the most populated metropolitan areas. We found that overall greenness increased across time with a 2.86% increase from 2000 to 2010 to 11.11% from 2010 to 2019. These results can be used to support CAPs by providing a quantitative assessment to the impact local greening initiatives can have on mortality. Urban planners and local governments can use these findings to calculate the co-benefits of local CAPs through a public health lens and support policy development.

## Introduction

Approximately half of the world's population resides in urban areas and this number is expected to increase 12–20% in the coming decades due to population growth and rural-urban migration, which will result in a doubling in the number of mega-cities^1^. As of 2016, roughly 80% of the United States (US) population live in urban areas (1). Increased urbanization leads to the conversion of landscapes from natural (i.e., grass and trees) to human-made materials (i.e., asphalt and concrete), while urban migration decreases connectivity between nature and humans (2). Wilson's *Biophilia hypothesis* suggests humans have evolved to have an affinity to nature, including plants, animals, and water bodies (3). There is an inherent value placed on exposure to nature and the benefits associated with health, specifically related to exposure to greenness (4, 5).

The Normalized Difference Vegetation Index (NDVI) is the most commonly used metric to estimate the quantity of green vegetation in recent epidemiological studies (5–10). There is evidence to support that NDVI is correlated with actual greenness on the ground and therefore is a useful proxy for greenness exposure (8). Greenness has used to estimate the mean density of green vegetation in a specific spatial domains such as a census tract and zip codes (5). Greenness has been shown to impact mortality risk though direct and indirect pathways such as increasing physical activity and social cohesion, reducing stress, mitigating the effects of heat and noise, and filtering out air pollutants (4, 5, 9, 11). These benefits associated with greenness exposure have been studied in the US (9), Switzerland (6), Canada (7, 11, 12), the United Kingdom (13), and other countries (14, 15). For example, in a nationwide Swiss cohort study of 4.2 million adults with 7.8 million follow-up years, investigators concluded that individuals living in greener areas had lower levels of mortality (12). There is substantial evidence on the relationship between mortality and greenness in the literature including meta-analyses and systematic reviews (4, 5, 15). Studies have also found this protective association in older populations with proximity to green space associated with increased longevity (16–18).

While there is robust literature suggesting that living in greener areas has an influence on mortality, we still lack information on how shifts in greenness distribution across the US could affect death rates. Health impact assessments (HIA) can address this gap by estimating the impact on mortality if sustainability goals and greening initiatives were successful. To our knowledge, only one recent HIA has been conducted which estimated yearly premature mortality for adults associated with the projected changes in tree canopy cover for Philadelphia between 2014 and 2025 (19). In this study, we conducted a nationwide quantitative HIA focused on the reduction of all-cause mortality for adults aged 65 and older residing within large metropolitan areas in the US if greenness were increased at the census tract level across three distinct time periods: 2000, 2010, and 2019.

## Methods

### Study Population

**Figure 1:**
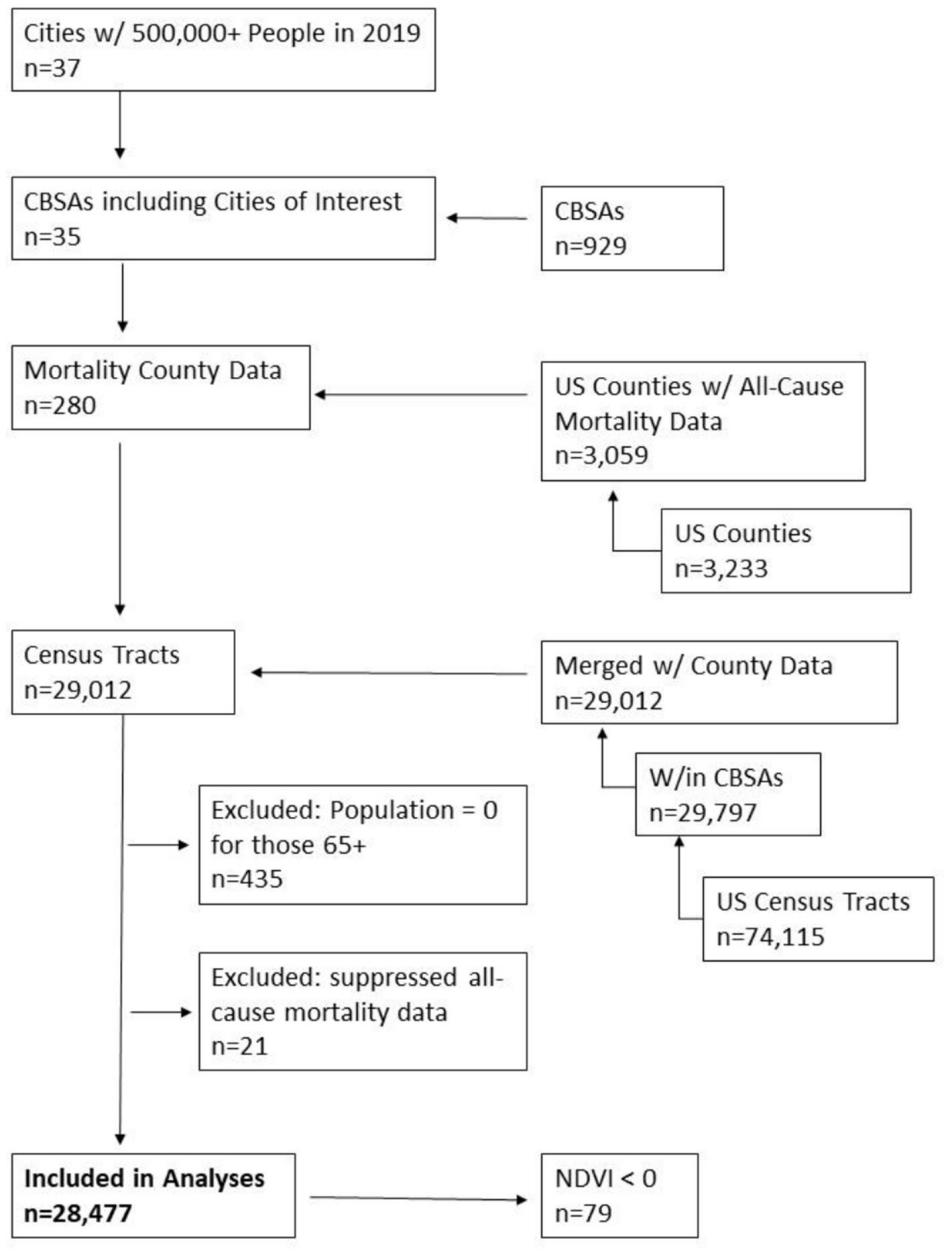
Flow chart of data.

Our study domain included all US metropolitan areas that contained a city with a population >500,000 in 2019 (*n* = 37 cities). The US Census defined core-based statistical areas (CBSAs) were used as boundaries for the metropolitan extent to identify census tracts for inclusion in our study as the unit of analysis (*n* = 29,797). Census tract population and age groups were retrieved from the US Census American Community Survey (ACS) 5 year estimates (20). Census tracts were excluded if: (1) had a population of 0 for those 65 and older, (2) average NDVI was <0, or (3) mortality data were suppressed (see Figure 1 for data flow chart). The final dataset included 28,477 census tracts after exclusion criteria.

### Mortality Data

All-cause (International Classification of Disease codes: ICD-10: A00-Y89) annual mortality counts and rates for 2000, 2010, and 2019 were obtained from the Centers for Disease Control and Prevention (CDC) WONDER data portal on a county level (21). Data were restricted to those 65 years and older. There were a total of 280 counties within the CBSA study areas (Figure 1). Six counties (representing 2%), and 21 census tracts (<1%) of the study population, were excluded from the analysis due to data suppression by CDC WONDER, which suppresses mortality counts below and equal to 9 persons for privacy purposes (see Supplementary Table 1A for example of data suppression by age-group for 2010 mortality data) (22). A total of 274 counties with annual mortality counts were included in the final analysis.

### Greenness Exposure

**Figure 2:**
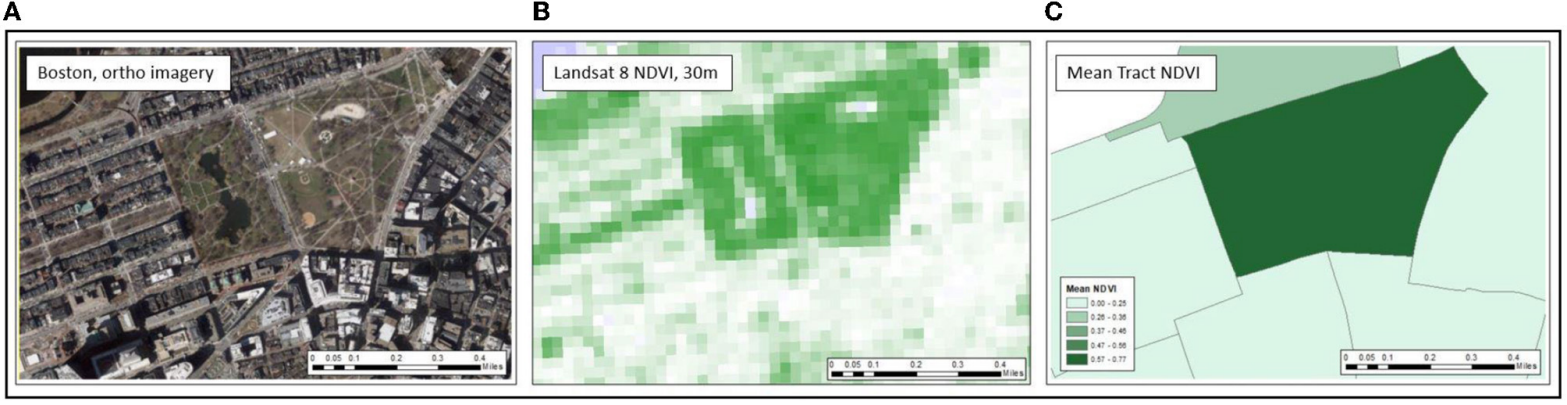
Representation of assigning 30 m NDVI to Census tract in comparison to orthoimagery. Panels run left to right. **(A)** Orthoimagery of Boston Commons, Boston, MA. **(B)** Raw NDVI image at 30 m resolution where darker green represents more green vegetation. **(C)** Mean tract NDVI aggregated from 30 m resolution.

NDVI was used to estimate the mean amount of greenness in census tracts. NDVI was derived from Landsat satellite imagery captured every 16 days at a 30 m resolution and calculated as the ratio of near-infrared to visible light providing an objective measure of vegetation quantity. NDVI was downloaded from Google Earth Engine (GEE) (23) between April and September for each of the three distinct time points, 2000, 2010, and 2019. The temporal range from April to September was used to identify the overall maximum greenness of an area and to minimize the amount of tracts missing NDVI due to cloud cover. We selected images with the least amount of cloud cover during the time period and all tracts within the study area were assigned a mean NDVI value using GEE. Figure 2 offers a visual of how NDVI 30 m data and mean tract level NDVI compares to a true color image (orthoimagery). Tract boundaries that extended into large water bodies were cropped to follow coast/shorelines to reduce misclassification as water features create negative NDVI values.

### Greenness-Mortality Hazard Ratio

To estimate the potential health benefit of increasing greenness at the census tract level, we used the hazard ratio (HR) from Rojas-Rueda et al. (24), a recent meta-analysis that examined the relationship between greenness and all-cause mortality. In brief, the authors analyzed data from nine longitudinal studies that, when combined, totaled 8,324,652 individuals from seven countries. The majority of studies relied on Landsat derived NDVI. The pooled HR for all-cause mortality per 0.1 unit increase of NDVI was 0.96 (95% CI: 0.94–0.97) (24). We applied this hazard ratio in our health impact assessment to calculate potential lives saved with a 0.1 unit increase in NDVI at the census tract level. An NDVI unit change of 0.1 represented approximately a 0.1 standard deviation change in census tract mean NDVI in our study domain.

### Statistical Analysis

County, tract, and NDVI data were all merged and analyzed in SAS 9.4. Age-adjusted county (Equation 1) and Census tract mortality rates (Equation 2) and predicted mortality reduction using the additive inverse of the HR (Equation 3) were estimated for 2000, 2010, and 2019. The inverse of the hazard ratio estimated the number of deaths reduced.


(1) $$\begin{array}{r} \frac{Deaths_{County\left(CDC\right)}}{Population_{County\left(CDC\;\right)}} \end{array}$$

(2) $$\begin{array}{r} Population_{Tract}*\frac{Deaths_{County\left(CDC\right)}}{Population_{County\left(CDC\;\right)}} \end{array}$$

(3) $$\begin{array}{r} \left(Population_{Tract}*\frac{Deaths_{County\left(CDC\right)}}{Population_{County\left(CDC\right)}}\right)*\left(1-HR^{*}\right) \end{array}$$

^*^where HR = protective Hazard Ratio from Rojas-Rueda et al. (24).

A sensitivity analysis was conducted to examine if using a single NDVI 16-day average from the month of July produced meaningfully different HIA estimates compared to the wider temporal range (April-September) that was used in the primary analysis for 2019. To determine if our main NDVI results differed from those in our preliminary analysis we conducted a Wilcoxon signed-rank test comparing tracts that were assigned both a seasonal and July greenness value.

## Results

**Figure 3:**
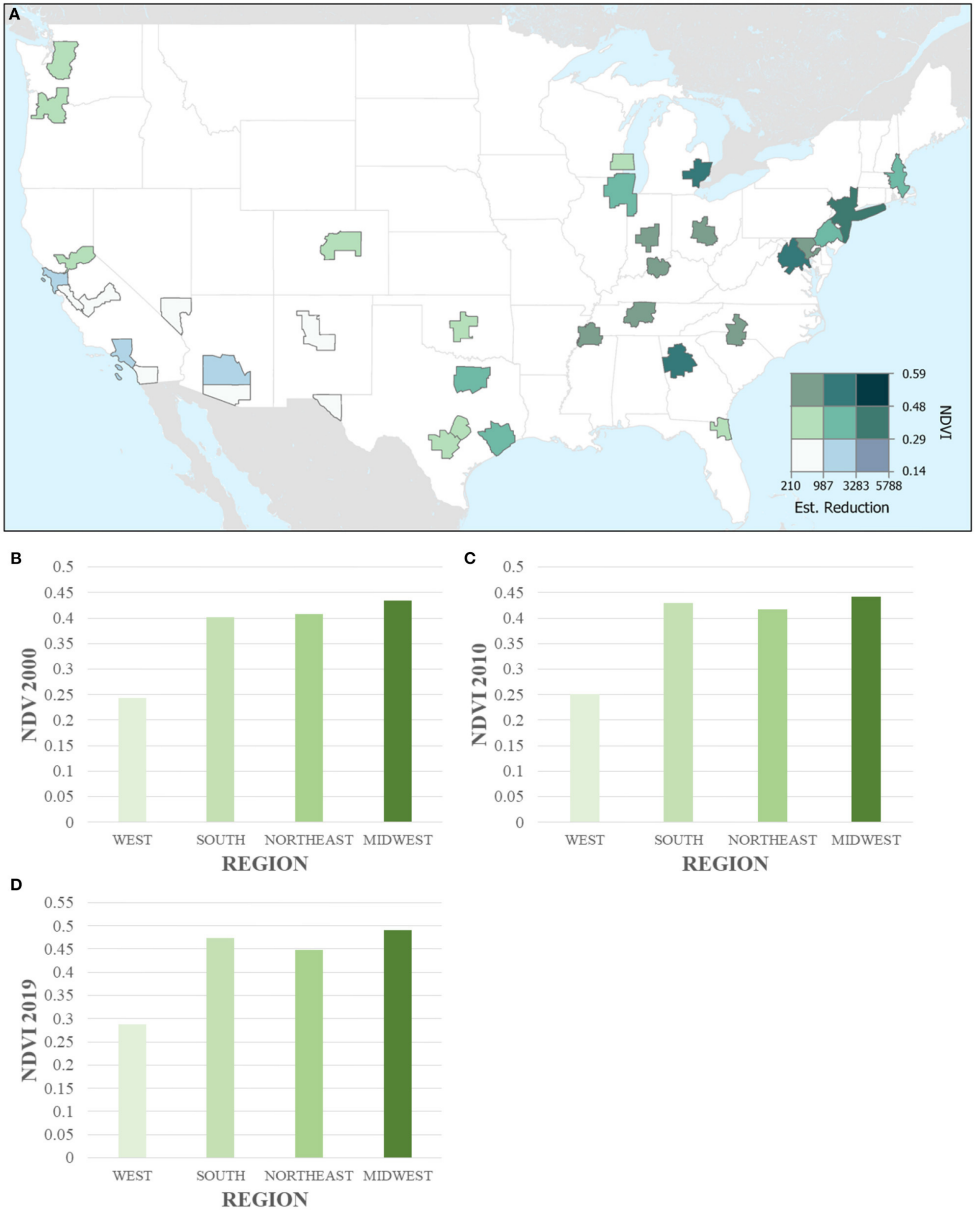
Greenness and All-Cause Mortality Reduction for 2019 **(A)** Greenness (NDVI) and absolute estimated reduction in all-cause mortality across 35 CBSAs (aggregated up from 28,477 census tracts). CBSAs are represented by polygons. **(B–D)** Bar charts of greenness (NDVI) in 2000, 2010, and 2019 across the four regions in the US (aggregated up from census tracts).

Our study domain included 35 CBSAs with a total estimated population between 17,396,014 in 2000 and 26,195,614 in 2019 for those 65 years and over. This population represents ~35% of the total US population 65 and over in across all three time points (20). Total deaths among those 65 and older were approximately 35% of the total deaths in for all time periods. Overall, mean census tract greenness ranged from 0.35 (SD: 0.16) to 0.40 (SD: 0.17) from 2000 to 2019. Looking more broadly at the regions within the US, the South had the lowest mean NDVI across all three time points (between 0.24 in 2000 and 0.29 in 2019); whereas the Midwest had the highest mean NDVI values for all time points (0.43 in 2000 to 0.49 in 2019) (Figure 3). The highest mean NDVI for 2000 and 2010 was 0.53 and 0.54 in the Charlotte-Concord-Gastonias metropolitan area in North and South Carolina. The highest NDVI for 2019 was 0.58 in the Nashville-Davidson-Murfreesboro-Franklin area in Tennessee. The lowest mean NDVI for all three time points was 0.11 (2000 and 2010) and 0.15 (2019) in the Las Vegas-Henderson-Paradise metropolitan area in the state of Nevada.

We quantified the potential reduction in all-cause mortality at the census tract level for a 0.1 unit increase of NDVI to estimate the predicted reduction in deaths of those 65 and older. In total, we estimated there would have been ~34,000 fewer deaths in 2000, and 2010, and 38,000 fewer deaths in 2019 if greenness were increased by 0.1 in all census tracts (Table 1). The estimated reduction in deaths per 10,000 for a population of 65 and over was highest in 2000 where 20 deaths per 10,000 would have been reduced. Over the following two time points, 2010 and 2019, this reduction decreased from 17 to 15 per 10,000. Table 1 displays the descriptive statistics for population, greenness exposure, and estimated reduction across the three time points. Overall, tract greenness increased across the three time points with a 2.86% increase from 2000 to 2010 and an 11.11% increase from 2010 to 2019. Looking specifically at the different regions across the US, we found that greenness increased across all regions with the largest increase seen in the South from 0.40 in 2000 to 0.47 in 2019.

**Table 1:** Summary statistics of population, exposure, and estimated reduction in all-cause mortality in study area.

**Year**	**Total population**	**Population ≥65**	**All-cause mortality ≥65**	**NDVI (SD)**	**Est. reduction (range)**	**Est. reduction per 10,000**
2000	113,508,720	17,396,104	634,665	0.35 (0.16)	34,846 (26,135–52,270)	20
2010	125,417,712	20,109,574	630,948	0.36 (0.16)	34,080 (25,560–51,120)	17
2019	133,273,276	26,195,614	753,300	0.40 (0.17)	38,187 (28,640–57,281)	15

We conducted a sensitivity analysis to examine the potential for exposure misclassification when using a single NDVI time period compared to a wider temporal average greenness. Health studies have calculated greenness exposure in similar ways, by choosing the greenest months, seasonal average, or summer months (12, 16). We used NDVI from 1 month (July 2019) to compare with our seasonal greenness method. Due to cloud cover approximately 0.3% of tracts for the month of July had NDVI values that were not useable (negative values).

To determine if our main results differed from those in our preliminary analysis we conducted a Wilcoxon signed-rank test in SAS 9.4 comparing tracts and CBSAs that had were assigned both a seasonal and July NDVI value (*n* = 38,391). The mean tract difference between these values was 0.03 (with a range of −0.20–0.75) units meaning that overall, seasonal NDVI values were higher than those in July. This difference was meaningful in the Wilcoxon signed-rank test with an alpha level of 0.05. Aggregating this analysis up to the CBSA-level, the average difference between seasonal and July NDVI was 0.03 (with a range of −0.05–0.46) and the total estimated reduction in all-cause mortality that would be missed if using July NDVI only was 127 deaths. The potential health impact was also compared, we estimated that by using the July NDVI values the total reduction in mortality 38,060 (28,545–57,091). This is due to 86 tracts being excluded in the July analysis due to unusable NDVI values. See Supplementary Figure 1 for the difference in NDVI aggregated up to the CBSA-level.

## Discussion

This study estimated the potential for reductions in mortality resulting from increased greenness in 35 metropolitan areas in the US. We found that 34,080–38,187 deaths in people 65 and older from 2000 to 2019 could have been reduced if NDVI increased by 0.1 units in all of the urban census tracts within the study. Overall, approximately 15 to 20 deaths per 10,000 in populations aged 65 and over could be reduced with an increase in NDVI. Estimated reduction in all-cause mortality, absolute and per 10,000 in total population, varied based on year. We found that greenness has generally increased across the 35 metropolitan areas in our study by 2.86 % from 2000 to 2010 and 11.11% from 2010 to 2019 with an average of 0.35 in 2000, 0.36 in 2010, to 0.40 in 2019.

Focusing on those 65 and older enabled us to quantify the potential reductions in mortality looking at a generally vulnerable population (18). This inverse relationship has been supported in previous studies looking at the direct and indirect pathways greenness can influence mortality (16–18). Most recently, a large cohort study conducted in Hong Kong focused on the indirect association between greenness and respiratory mortality due to air pollution, found that elders living in low greenness areas had a higher rate of mortality than those living in higher greenness areas (17). There is also evidence that suggests greenness can increase physical activity, such as walking, and in turn have a protective effect on mortality (18, 25).

NDVI can be a coarse measure of greenness and does not convey quality, accessibility, or health of vegetation and is different from greenspace exposure. Greenspace consists of open, undeveloped land with natural vegetation (26). In more general terms, greenspace can be defined as areas that include some type of “green” vegetation, such as lawns, shrubs, parks, trees, and forests. NDVI does not allow for the differentiation between an agricultural field and a park but it does provide an objective measure of the amount of vegetation within a certain area. This lack of consistency can be a problem for policymakers since an increase in NDVI can look very different between census tracts. However, Rhew et al. (7), reported that NDVI is a useful measure of neighborhood greenness and was strongly correlated with ground truths based on psychologists' ratings (8). In addition, NDVI still remains a widely used exposure metric for greenspace and greenness in large-scale epidemiologic studies and a recent HIA (4, 5, 10, 19). We found that the 0.1 unit increase in NDVI is realistic at both the tract and CBSA-level. We found that 409 tracts between 2000 and 2010 and 731 tracts between 2010 and 2019 increased their greenness by at least 0.1 units. This assumption is further supported by a recent national study that concluded NDVI can change by almost 0.7 units over a decade (27). Other studies have also used this 0.1 unit increase (9, 28). We are aware that NDVI and the potential to increase NDVI is not the same across the US due to varying climate zones. Such that greening initiatives in Denver, CO should not be the same as those implemented in Boston, MA since these metropolitan areas do not have similar climates (i.e., temperature, humidity, etc.). Therefore, local urban climates should be taken into consideration when using these results to support greening initiatives.

We also recognize that there may be a lack of generalizability within our study by focusing on CBSAs that include a major city. Approximately 80% of the US population living in urban areas as of 2015, therefore we believe that by focusing primarily on metropolitan areas we are using a representative sample to quantify the benefits of increased greenness (1, 20). Although, CBSAs may not always be representative of their regions. Another limitation of this HIA, is that it assumes a linear exposure-response function between greenness and mortality. Although this assumption is supported by previous literature, other studies have shown a potential non-linear relationship (7, 9, 16). The dose-response function used in our methods from the Rojas-Rueda et al. (24) meta-analysis assumes linearity (24). This assumption implies that the effect of greenness is the same in both low and high income neighborhoods (i.e., no effect modifiers), between urban and rural areas, and across race/ethnicity groups, which may not be the case. This study only focuses on major metropolitan areas. However, the studies within the meta-analysis controlled for socioeconomic and other relevant variables in their analysis (24). If exposure to greenness has a non-linear relationship with mortality then we could be under predicting the mortality reduction in lower NDVI tracts and over predicting mortality reduction in higher NDVI areas. There could be greater benefits to improve green infrastructure in areas that are lower on the NDVI scale (i.e., 0.1–0.2) than at the higher end (i.e., 0.7–0.8). More studies on the relationship between greenness and mortality would allow for increased likelihood of incorporating the true exposure-response relationship in future studies. Another limitation of our study is that greenness exposure is estimated at the census tract and downscaled county-level mortality which can introduce exposure misclassification from ecological bias. The distribution of greenness in a metropolitan area may not be equitable and therefore we are aware that downscaling deaths from county to tract is a limitation. Future studies should utilize higher resolution mortality data that could shed light onto this limitation. Lastly, there is the potential for survival bias in because we restricted the population to those who have made it to 65 years of age and older.

Despite its limitations, this study has many strengths. One strength of our study is that it looks at the longitudinal change of greenness distribution across two decades in the largest metropolitan areas. Another strength, is that we assessed the estimated benefits of nationwide greening using an exposure-response function from a meta-analysis to give tangible numbers to policy makers. Using publicly available data, we were able to estimate one possible impact of greening cities. Although greening across all cities may not be realistic due to differing climates and water resources, sustainability and climate action plans are already setting greening goals such as increasing tree canopy and green infrastructure (29). By quantifying the potential lives saved with an increase in greenness, urban planners and policy makers can use this assessment to show the co-benefits of increased greenness for health and climate mitigation. The purpose of our study is not a validation but rather quantifying the potential for reducing all-cause mortality, based on results from peer-reviewed research.

## Conclusion

In conclusion, by increasing tract-level NDVI by 0.1 units across the largest metropolitan areas in the US, we could prevent over 34,000 deaths according to 2000, 2010, and 2019 data. Using greenness data from these time periods, we found that increasing greenness (NDVI) by 0.1 units is possible keeping in mind that greening may not be realistic in some urban areas due to environmental or climatic factors. Given the potential health benefits of greenness and greenspaces, urban planners and policy makers can use findings from this study to support sustainability and climate action plans^2^ that call to increase vegetation, such as the New York City Million Trees NYC or Green Heart Louisville, in Louisville Kentucky (30, 31). Our results presented here support these policies by providing a public health lens to greening cities.

## Data Availability Statement

Publicly available datasets were analyzed in this study. This data can be found at: All-Cause Mortality Data, CDC WONDER Portal: https://wonder.cdc.gov/ucd-icd10.html NDVI Data, Landsat 8 30 m, Google Earth Engine: https://developers.google.com/earth-engine/datasets/catalog/LANDSAT_LC08_C01_T1_32DAY_NDVI.

## Author Contributions

PB, KL, PJ, PK, and MJ generated the project idea, contributed to data interpretation, and review and revision of the report. PB, KL, MJ, and PJ collected the data. PB and KL assessed and verified the underlying data. PB did the analysis and had the primary responsibility of writing the manuscript. All authors contributed to writing the manuscript.

## Funding

This work was funded by the National Institutes of Health (1K99AG066949-01, NIH; P50 MD010428, T32-HL098048, R00CA201542, and R01HL150119), US Environmental Protection Agency (EPA; 83615601), National Institute of Environmental Health Sciences grant (NIEHS; T32 ES014562), and National Science Foundation (NSF NRT DGE1735087).

## Author Disclaimer

This manuscript's contents are solely the responsibility of the grantee and do not necessarily represent the official views of the USEPA. Further, USEPA does not endorse the purchase of any commercial products or services mentioned in the publication.

## Conflict of Interest

The authors declare that the research was conducted in the absence of any commercial or financial relationships that could be construed as a potential conflict of interest.

## Publisher's Note

All claims expressed in this article are solely those of the authors and do not necessarily represent those of their affiliated organizations, or those of the publisher, the editors and the reviewers. Any product that may be evaluated in this article, or claim that may be made by its manufacturer, is not guaranteed or endorsed by the publisher.

## Footnotes

^1^ https://population.un.org/wup/publications/files/wup2014-report.pdf

^2^ http://www.seattle.gov/documents/departments/ose/2013_cap_20130612.pdf

## Supplementary Material

Supplementary Figure 1 Difference in average CBSA greenness between July 2019 and seasonal 2019 NDVI data aggregated from tract-level. *Areas in symbolized by red indicate metropolitan areas that had a higher NDVI captured in July compared to the seasonal average between May and September. Areas in Green and Blue indicate those that had higher seasonal averages compared to July.

Supplementary Table 1A Suppressed 2010 Mortality Data (*n* = 280).

## Abbreviations

CAPs: climate action plans
NDVI: normalized difference vegetation index
US: United States
HIA: health impact assessment
CBSAs: core-based statistical areas
ACS: American Community Survey
CDC: Centers for Disease Control and Prevention
GEE: Google Earth Engine
HR: hazard ratio
SD: standard deviation.
