# Supplementary figures and images for: Benefits of Increasing Greenness on All-Cause Mortality in the Largest Metropolitan Areas of the United States Within the Past Two Decades
Source: Front Public Health. 2022 May 10;10:841936. doi: 10.3389/fpubh.2022.841936 (PMC9127575; doi:10.3389/fpubh.2022.841936)

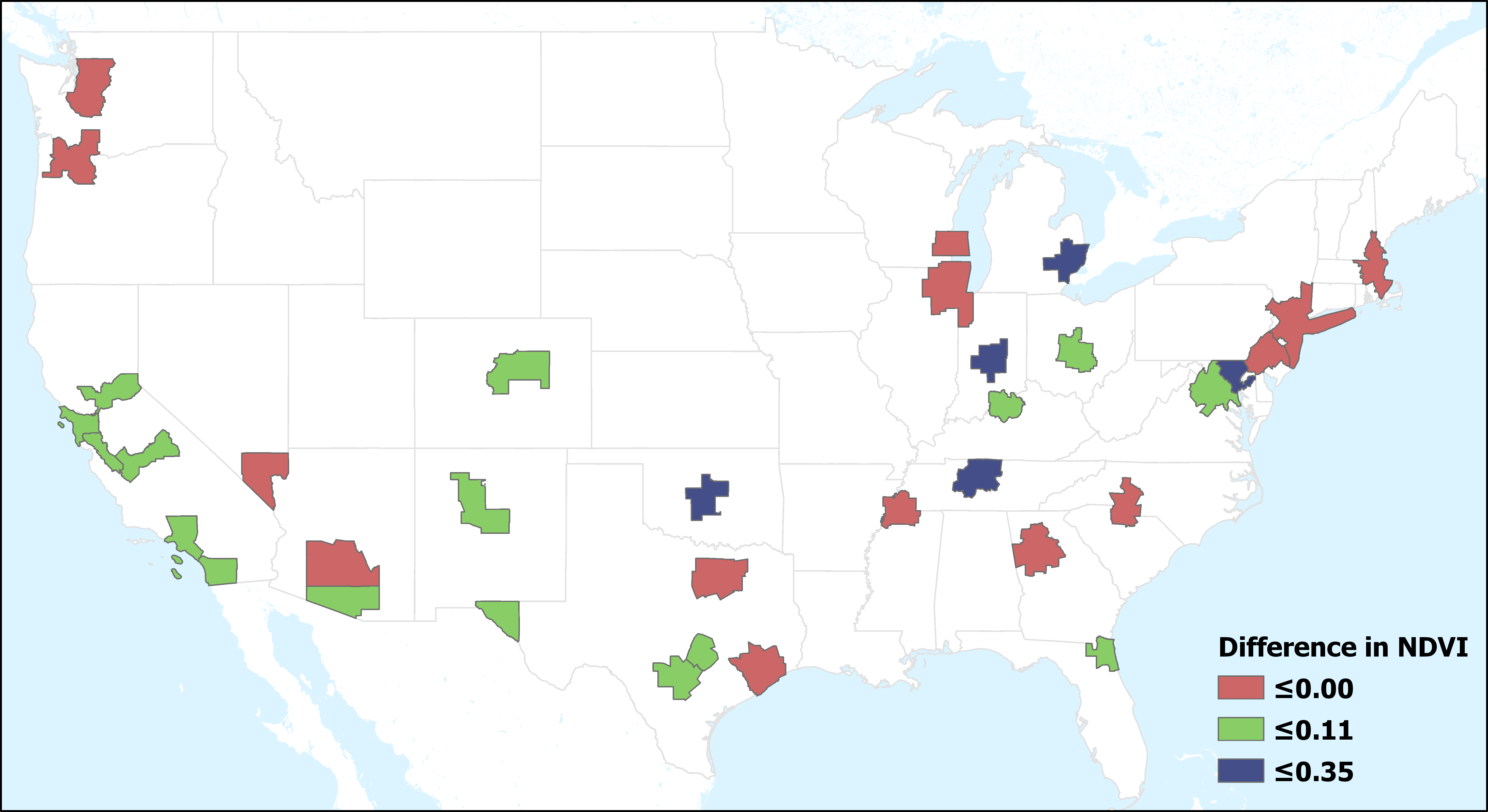

Supplement: Supplementary Figure 1 — Difference in average CBSA greenness between July 2019 and seasonal 2019 NDVI data aggregated from tract-level. *Areas in symbolized by red indicate metropolitan areas that had a higher NDVI captured in July compared to the seasonal average between May and September. Areas in Green and Blue indicate those that had higher seasonal averages compared to July. [file Image_1.TIF]
