# Supplementary material for: Benefits of Increasing Greenness on All-Cause Mortality in the Largest Metropolitan Areas of the United States Within the Past Two Decades
Source: Front Public Health. 2022 May 10;10:841936. doi: 10.3389/fpubh.2022.841936 (PMC9127575; doi:10.3389/fpubh.2022.841936)
Supplement: Supplementary Table 1A — Suppressed 2010 Mortality Data (n = 280). [file Table_1.DOCX]

| Supplemental Table 1A. Suppressed 2010 Mortality Data (n=280) | | |
| --- | --- | --- |
| Age-Group | # Counties | Percent of Total Counties |
| 25-34 | 84 | 30 |
| 35-44 | 45 | 16.1 |
| 45-54 | 10 | 3.6 |
| 55-64 | 2 | 0.7 |
| 65-74 | 1 | 0.4 |
| 75-84 | 2 | 0.7 |
| 85+ | 2 | 0.7 |
